# Simulosophists: evolving the professional identity of simulation practitioners

**DOI:** 10.1186/s41077-026-00417-y

**Published:** 2026-03-16

**Authors:** Griselda Gonzalez-Caminal, Laura Castillejos-Gallego, Carmen Gomar-Sancho

**Affiliations:** 1 Global Research on Wellbeing (GRoW) Research Group, Blanquerna School of Health Sciences, Universitat Ramon Llull, Barcelona, Spain; 2 Institut de Recerca i Innovació en Ciències de la Vida i de la Salut de la Catalunya Central. IRIS-CC. Vic, Barcelona , Spain; 3 BSN and Simulation Practitioner, Dakar, Senegal; 4 Research Group on Transformative Innovation and Simulation (GRITS), University of Vic-University of Central Catalonia, Manresa, Spain

**Keywords:** Simulation, Professionalism, Facilitator, Professional identity, Psychological safety

## Abstract

Simulation educators frequently cultivate psychological safety and reflective practice within simulated environments, yet these same values often fail to translate into their broader professional contexts as members of teaching department or healthcare teams. This inconsistency—between what is modelled in simulation and what is lived in everyday work—reveals a deeper issue of professional dissonance that is not fully addressed by existing simulation standards and codes of ethics, which largely focus on conduct within educational activities rather than in wider workplace cultures. Moreover, many simulation practitioners “wear multiple hats,” simultaneously inhabiting roles as clinicians, educators, and leaders, which can blur boundaries and expose tensions between espoused values and organizational hierarchies. This conceptual paper proposes the concept of the simulosophist—a simulation practitioner who not only teaches but lives on the foundational principles of simulation: ethical integrity, reflection, and psychological safety-across roles and contexts-and argues that the evolution toward this identity requires coordinated action at individual, institutional, and disciplinary level. The simulosophist bridges the gap between simulated and real-world practice, modeling coherence across roles and contexts. By embracing this multi-level perspective and this proposed professional identity, simulation practitioners can advance simulation not merely as a pedagogy, but as a culture of integrity and professional practice that is consistently enacted beyond the simulation room.

Starting point: After facilitating a simulation session in a welcoming environment that encourages open dialogue and diverse viewpoints, the facilitator leaves the room feeling that the principles of psychological safety and continuous learning emphasized during the session do not persist in the other work settings in which they are involved, either clinical or not clinical. The authors have experienced this themselves as facilitators and simulationists, and from these experiences, they have drawn several reflections.

## The simulosophist: expanding the concept of the simulation practitioner

As simulation becomes foundational in healthcare education and systems improvement, it is essential for the identity of those who design and lead it to evolve as well. The term “simulationist” is used in its historical and broad acceptance sense to describe professionals who design, deliver, and facilitate simulation-based educational activities and programs. As noted by Kardong-Edgren, the term has evolved and might differ and be used variably across contexts to refer to simulation educators, technicians, and clinical faculty engaged in simulation practice. In this paper the authors have agreed on using the term simulation practitioner to denote this foundational professional role. While simulation standards and codes of ethics [1–5] define professional conduct during educational activities, they seldom address how these values translate into workplace behaviors and struggle to maintain it in daily interactions [6, 7]. Therefore, the term “simulationist,” as currently used, may need to be expanded [8, 9]. The authors propose the neologism “simulosophist” (from *simulation* + *philosophy*) as the aspirational archetype of a simulation practitioner who not only masters simulation-based education methods and techniques but also models reflective, ethical, and psychologically safe practice across all domains of their work.

*simulosophist (n.): A simulation professional who exemplifies and integrates the core values of simulation—ethical integrity*,* reflection*,* and psychological safety—across all domains of their professional and personal life.*

One question guided our discussion in presenting this proposal: “Why is a new identity needed?”

### The need for a new professional identity

In recent years, simulation has been increasingly implemented in a broad range of educational contexts and disciplines. With this expansion, the methodological foundation has become more important than the technological one [1].

As simulation methodology relies on relational processes such as facilitated reflection, collaborative learning and feedback and, the creation of a safe environment cannot be separated from the professional behaviours and values of those who enact it. Therefore, an increased focus on methodology inevitably foregrounds questions of professional identity and integrity, rather than solely technical competences.

Professional integrity entails maintaining a coherent set of values, behaviors, and ethical principles that guide professional identity and practice [10]. It aligns personal and institutional goals to ensure responsible fulfillment of professional duties. At the same time, it preserves courtesy, respect, and collegiality in interactions with colleagues, students, and institutional members.

The International Nursing Association of Clinical Simulation and Learning (INACSL) – as well as other simulation-related organizations and bodies – reiterates the need for professional training and ethics development to apply simulation ​​ [2] and provide valuable guidelines to support the design of high-quality simulation activities [3, 4, 11]. Aligned with that, the simulation code of ethics focuses on the competencies that simulation practitioners must apply to all training and/or teaching activities in which they participate [5]. As stated in the code of ethics, it “is intended to ensure that healthcare simulation practitioners perform all activities to the highest ethical standards across the globe” [5]. The code, however, does not consider the professional integrity of the simulationists – i.e., ethical principles and values in the daily workplace environment outside the simulation room.

Outside the simulation room is used here deliberately to encompass a broader range of professional contexts, including educational settings, simulation centers, institutional meetings-either clinical or non-clinical-and interactions among peers who could be themselves simulation practitioners. The dissonance described is therefore not limited to the transition between simulation and clinical practice, but also emerges at multiple levels among facilitators and between simulation practitioners and in exchanges with colleagues outside the field of simulation.

This gap raises an important ethical question: to what extent do simulation principles constitute a professional responsibility beyond the simulation room? While environmental, hierarchical, and cultural constrains may limit this translation of the principles into other daily practices and activities, the tension between espoused values in simulation and enacted behaviours in the workplace highlights a deeper challenge of professional identity rather than ethical guidance alone.

Professional identity in simulation is shaped not only by formal ethical codes but by a dynamic and interdependent set of factors that operate as a system. These include organizational culture, professional hierarchies, role multiplicity, institutional expectations, and everyday workplace behaviours. The persistent misalignment between simulation values and real-world practice suggests that technical competence and ethical frameworks, while necessary, may be insufficient to support coherent professional identity across contexts [12, 13].

### Why are we not there yet? Tensions limiting the evolution of simulation practitioners

This inconsistency between simulation activities and the facilitator’s workplace activities may stem from a lack of professional integration. The current professional identity is often narrowly defined by technical roles. Especially when considering simulation healthcare education, simulation practitioners performing roles as learning designers and facilitators, crafting scenarios that mimic real-life challenges, encouraging decision-making and reflection, and providing safe environments for competence advancement and process and system development [5].

**Fig. 1:**
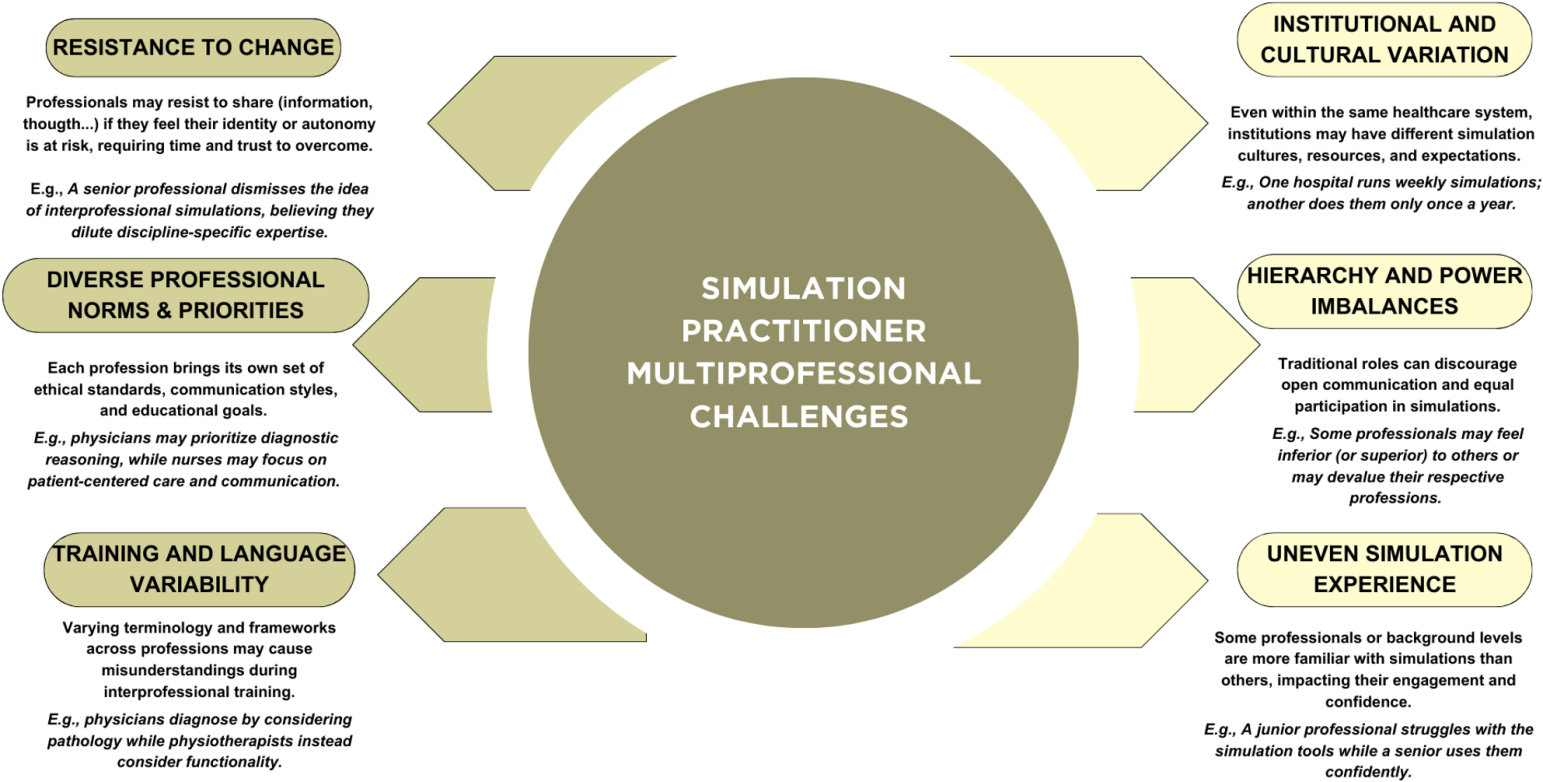
Multiprofessional challenges: persistent structural conditions to recognize and navigate at personal and institutional levels

The point becomes more complex here, as simulation practitioners most of the times work alongside colleagues from diverse professional backgrounds, often occupying different roles such as clinicians and educators [14]. Without a model of professional integrity -defined here as an aligned commitment to ethical conduct, reflective practice, respect for diverse expertise and psychological safety- these challenges may undermine collaboration and learning. As illustrated in Fig. 1, these factors are not merely individual barriers to remove but enduring structural conditions that can hinder the development of a cohesive simulation culture when core values are not consistently embedded and shared across professional identities. When these conditions go unrecognized or unaddressed, multiprofessional simulation teams risk reproducing hierarchical, soiled, or psychological unsafe practices that simulation methodology itself seeks to challenge.

Multiple studies point out that many teams remain in psychologically unsafe work environments [9, 15]; simulation teams might not be different. Amy Edmondson’s work [16] distinguishes psychological safety—a shared belief that a team is safe for interpersonal risk—from the idea of a “safe space,” which protects comfort rather than facilitating learning. Simulation methodology naturally integrates this concept through practices during the various phases of simulation, particularly during briefing and debriefing. Moreover, creating a “safe container” is both a commonly used term and an expected behavior [17]. However, these behaviors often fade once the session ends.

Implementing psychological safety requires intentional action, including open communication, non-judgmental feedback, and recognizing mistakes as learning opportunities. Maintaining these behaviors in high-pressure and hierarchical environments where performance and time constraints dominate can be challenging [8, 14]. Moreover, personal biases, past experiences, and interpersonal dynamics can further complicate efforts, making it hard for team members to trust that their vulnerability will not have negative consequences at their workplace [18].

This tension highlights a form of professional dissonance—simulation practitioners often advocate for psychological safety in learner settings while working within hierarchical, and sometimes psychologically unsafe, teams themselves. Such contrasts expose a gap between espoused and enacted values. As Dace et al. note, simulation professionals frequently “wear multiple hats,” blending their roles as clinicians, educators, and leaders, which can blur boundaries and challenge consistency in the application of psychological safety principles across contexts [14].

### How do we get there? Simulation as a culture of practice

Simulation methodology—from structured debriefings to safe container principles—provides a robust framework for fostering a professional culture. These methods function as behavioral scaffolds that can be adapted for use in team meetings, interprofessional collaborations, and leadership practices, rather than serving solely as instructional tools. In fact, these same methods are the ones that apply to the “clinical debriefing” for natural clinical teams [19, 20].

The principles of psychological safety, learning without harm, constructive feedback, and ethical integrity must become integral to simulation practitioners professional practice and behavior from the start of their training. Junior staff learn about culture and behavior by observing how their seniors behave in meetings, during stressful situations, and in conflict. This idea is consistent with Bandura’s social cognitive theory, which emphasizes that learning occurs not only through direct experience but also through observation and imitation of role models [21]. This is especially important for those responsible for training future professionals in simulation—whether through formal education or mentorship—should begin cultivating these values early.

In simulation, observational learning is as effective as participation [22, 23]. The simulosophist recognizes that psychological safety is not a performance confined to simulation activities, but a lived commitment. Consequently, novice simulation practitioners should be able to observe this consistently in both peer and senior role models.

Viewed in this way, simulation can be understood not only as an educational methodology, but as a cultural practice capable of shaping professional norms and values in everyday work environments [12, 13]. Debriefing becomes a model for performance reviews. Psychological safety becomes a foundation for innovation. Pre-briefing becomes a structure for inclusive meetings. In this evolved approach of the simulation practitioner, the core values of simulation are not left behind in the simulation room but intentionally adapted and carried into other professional contexts.

**Fig. 2:**
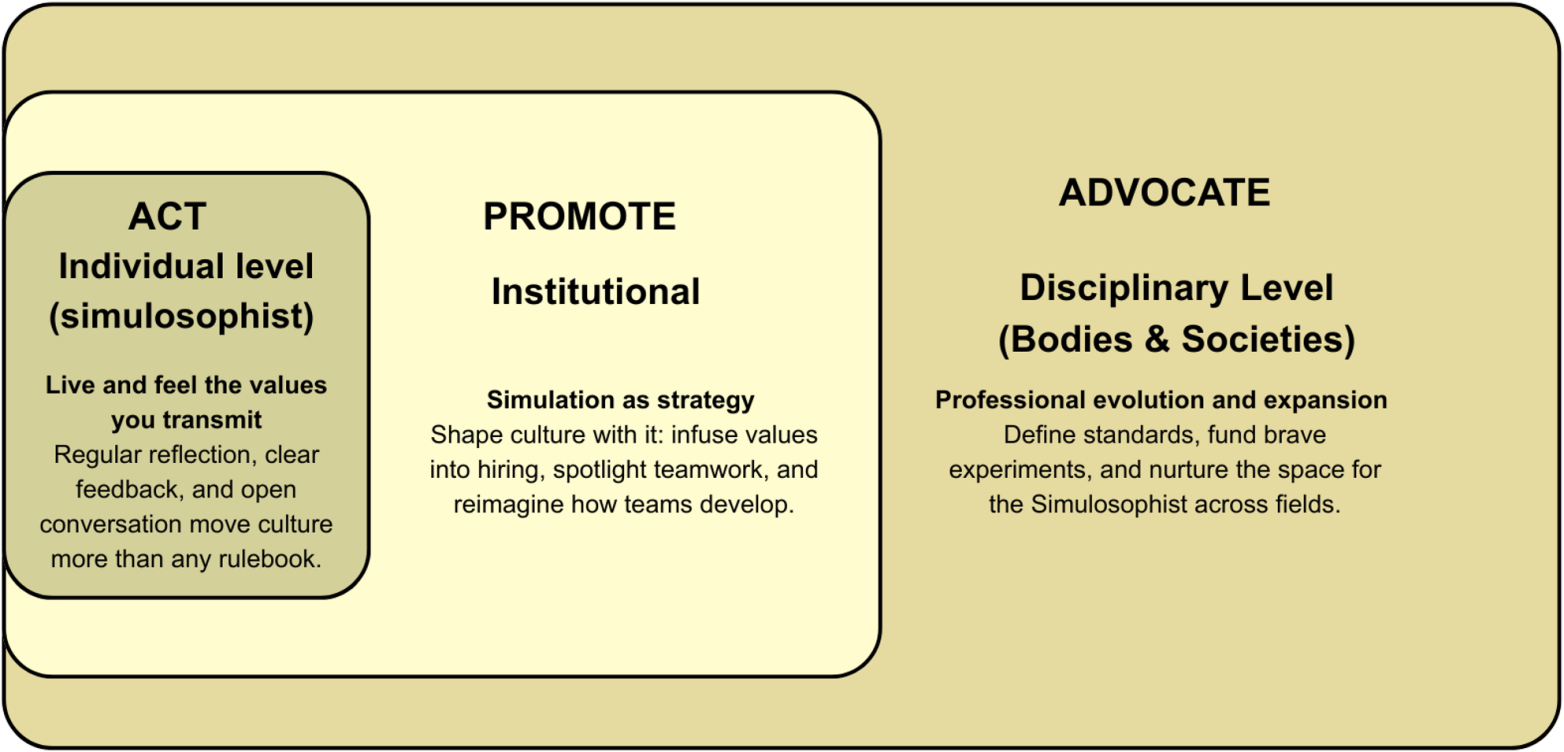
A three-level systemic model of professional evolution toward the simulosophist

The transition from simulation practitioner to simulosophist is both a personal journey and a professional evolution. The transformation toward this identity requires action at three levels (see Fig. 2) allowing to overcome the challenges mentioned in Fig. 1.

Figure 2 illustrates the progressive and interconnected levels involved in the professional evolution of simulation practitioners toward the simulosophist identity.

- Individual Level: At the individual level, the transition begins with intentional alignment between the values enacted in simulation and everyday professional behavior. Practices such as active listening, encouraging diverse opinions, and modeling vulnerability-well described within simulation facilitation literature-can be carried into routine professional interactions, including team meeting, feedback conversations, and moments of conflictFor example, simulation-driven debriefing approaches can be adapted as brief reflective check-ins following challenging meetings or clinical events, emphasizing curiosity, shared sense-making, and learning rather than judging. Similarly, pre-briefing principles-such as clarifying expectations, acknowledging uncertainty, and inviting and creating psychological safety-can structure conversations before collaborative work begins. As shown in prior work on psychological safety and debriefing practices [14, 15, 17], these behaviors, while small in scale, have a cumulative cultural impact when maintained over time. All those are simple habits that reinforce a shared culture of respect, openness and professional integrity as a lived practice rather than a stated ideal within simulation activities.
- Institutional Level: Simulation can function as a strategic cultural asset and vehicle to transform the institution rather than solely a training modality integrated into activities. Organizations that intentionally integrate simulation principles into routine processes- such as using structured debriefing formats in quality improvement meetings or to provide feedback for professional development-create coherence between educational values and organizational behavior. However, institutional-level inconsistencies may still arise between espoused values and formal structures. For example, certification processes or career advancement are often linked to indicators that fail to account for professional integrity and professionalism within their assessment frameworks. While written assessments or other academics accomplishments effectively evaluate conceptual understanding and academic career, they may be limited in capturing the relational, reflective, and ethical dimensions of practice that simulation itself seeks to promote. Embedding psychological safety skills as explicit leadership or work competency, recognizing reflective practice in performance evaluations, and legitimizing time for collective and collaborative learning are examples of how institutions can operationalize and formalize simulation values. Such practices align with existing literature on learning organizations and psychologically safe teams [14, 18] and indicate that the values promoted in simulation are both expected and transferable to everyday work transforming organizations [24].
- Disciplinary level (Bodies and Societies): Professional bodies, simulation societies, and networks play a critical role in legitimizing this expanded professional identity. By explicitly acknowledging leadership, ethical coherence and culture-shaping as a core competency of simulation practice, these organizations can support simulation practitioners who seek to embody simulation values and professional integrity beyond simulation settings. This includes revisiting competency frameworks, supporting scholarship on simulation as a cultural practice, or creating spaces for reflective dialogue about professional identity.

Reflection is a central axis of the equation. Reflection, in this sense, is not an end but a process that allows simulation practitioners and organizations to reorient behaviours, decision, and interactions toward more effective and ethical grounded outcomes. When embedded across all levels-from individual practice and institutional culture to disciplinary statement-reflection contributes to responsiveness, adaptability, and continuous pursuit of improvement. By linking insight with action, reflective practice supports efficiency not through control or compliance, but through expanded awareness, shared purpose, and emancipatory practices and professional integrity alignment in everyday work.

Actions can be initiated at any level, but it makes more sense when undertaken at a disciplinary level by professional bodies, societies and networks, which define the normative boundaries of practice establishing precise standards, articulating ethical expectations, and signal what forms of professional behavior are expected and valued. Without this disciplinary endorsement, efforts at an individual or institutional level could remain fragmented, informal, and vulnerable to local hierarchies and norms. Positioning the disciplinary level as the starting point of professional evolution does not negate bottom-up influence; rather, it created the conditions under which such influence can be sustained and where individuals can grow.

## Conclusion

The simulosophist represents an evolution of professionalism in simulation, where the principles of simulation are not only taught but also lived. This paper argues that the core principles of simulation-professional integrity, reflection and psychological safety-constitute a professional responsibility that extends beyond the simulation room and must be lived across all roles, contexts and professional interactions.

Transferability operates across levels in a joint responsibility: individuals adapt simulation principles in everyday interactions, institutions embed them into organizational processes, and professional bodies articulate and legitimize these expectations through standards and competency frameworks. We advocate for an explicitly articulated change within simulation standards to make it visible that expectations on simulation values extend to beyond educational activities.

Our position is informed by existing frameworks such as the INACSL Standards of Best Practice, the ASPiH Standards, and the Healthcare Simulationist Code of Ethics, which we view not as context-bound rules but as a transferable ethical commitment adaptable to everyday professional practice. Only by aligning simulation methodology, personal and professional integrity, and team culture can we truly live simulation, not just teach it.

## Data Availability

Data sharing is not applicable to this article as no datasets were generated or analysed during the current study.
